# Unprovoked Atypical Absence Status Epilepticus in a Patient With Allogeneic Stem Cell Transplantation

**DOI:** 10.7759/cureus.64842

**Published:** 2024-07-18

**Authors:** Sumona Banerjee, Maaria Chaudhry, Jonathan Brewer, Yongzhen Chen, Farid Khasiyev, Momina Soudagar Turkey

**Affiliations:** 1 Neurology, Saint Louis University School of Medicine, Saint Louis, USA

**Keywords:** myelofibrosis, allogeneic stem cell transplant, case report, older adults, status epilepticus, absence seizure

## Abstract

Absence status epilepticus (ASE) is a rare but treatable condition, and when present in older adults, it can be misinterpreted as encephalopathy or behavioral changes. Our case discusses a 63-year-old patient with myelofibrosis and allogeneic stem cell transplant with late-onset de novo status epilepticus. This case report adds to the rare body of literature discussing de novo ASE whose clinical presentation can be indistinguishable from other encephalopathic or behavioral conditions. Moreover, its occurrence during oncologic treatment warrants clinicians to be on the lookout for similar presentations and encourages future reports of this condition in association with similar therapies. This case report provides value to providers treating patients with similar oncologic therapies and highlights the need for ASE to be further studied as it is a possible rare complication of allogeneic transplantation of stem cells.

## Introduction

Absence seizures are a subtype of generalized seizures with an onset that manifests as an abrupt halt in current behavior and is usually accompanied by a blank facial expression and impaired consciousness referred to as “staring spells.” These seizures are uninterruptible, and patients often have automatism, such as eye fluttering and lip-smacking [1]. Typical absence seizures last around 10 seconds and may happen up to hundreds of times per day. These are generally diagnosed in childhood and rarely continue into adulthood [1]. According to the International League Against Epilepsy task force, emergency treatment for this condition should be initiated within 10-15 minutes [2]. Therefore, early identification of the symptoms is crucial for prompt intervention.

Typical absence seizures occur roughly in 12-18% of children with epilepsy; however, data on adult rates of absence seizure epilepsy are unknown [3]. In addition, there frequently is no postictal phase associated with typical absence seizures [1]. In contrast, atypical absence seizures often last longer than 10 seconds [1]. They also more frequently persist into adulthood from a history of typical absence seizures in childhood [1]. Additionally, atypical absence seizures may have localized repetitive body movements similar to that of a focal seizure and postictal confusion [1]. Both typical and atypical absence seizures are exemplified on EEG with a classical pattern showing generalized 3-Hz spike-and-wave discharges [4]. This waveform can help distinguish atypical absence seizures from focal seizures.

Similar to other focal and generalized seizures, absence seizures may become prolonged in a state of status epilepticus. Absence status epilepticus (ASE) is defined as a prolonged, generalized absence seizure lasting for at least half an hour but usually lasting hours to days [5]. ASE may also present with previously discussed automatisms and is defined as either “typical” in the setting of idiopathic generalized epilepsy or “atypical” in the setting of unclassified epilepsy [5]. Although the exact prevalence of ASE is unknown, there are only a few reported cases of ASE as they are often prevented with early detection in pediatric patients and placement on medications. When it comes to elderly individuals, diagnosing ASE can be challenging, as they are already past the typical age of onset associated with idiopathic generalized epilepsy. Moreover, this condition is sometimes mistaken for encephalopathy or cognitive impairment-related behavioral changes. In this article, we present a unique case of late-onset de novo ASE in a hematologic oncology patient.

## Case presentation

A 63-year-old woman presented to the Emergency Department with daily episodes of confusion and decreased responsiveness lasting up to 60 minutes for a total duration of 48 hours. Her past medical history was significant for myelofibrosis and allogeneic stem cell transplant two years prior to the current presentation, and she had been taking beclomethasone, posaconazole, and enoxaparin as post-transplant precautions. She did not have a significant neurological history, including no history of epilepsy, prior to presentation. During the interview, which lasted approximately 15 minutes, she was noted to have over 25 episodes of staring spells, all of which were distractible with strong physical and auditory stimuli. The cessation of these events was abrupt, and the patient did not have tongue biting, bowel or bladder incontinence, tonic-clonic movements, eye deviations, or other motor phenomena. She was noted to have awareness of the episodes and had no apparent post-ictal state. Neurological examination was normal and the patient was placed on EEG.

**Figure 1:**
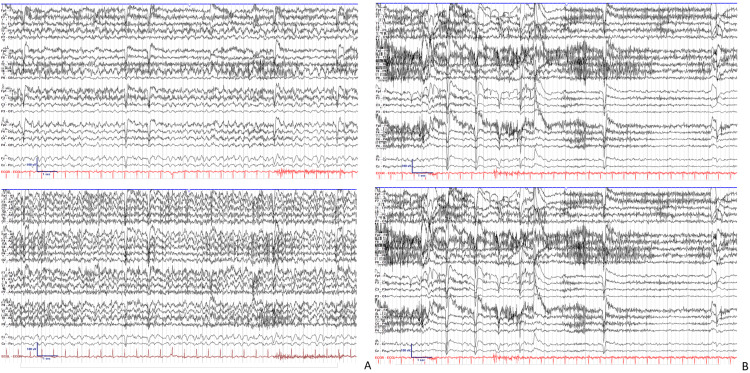
EEG of the patient in longitudinal and average referential montages showed (A) rhythmic 3-4 Hz with admixed sharp waves during wakefulness with associated clinical symptoms, consistent with electroclinical seizure, and (B) disappearance of midline rhythmic activity, which was associated with return to clinical baseline.

While admitted, the patient continued to experience intervals of blank stares, psychomotor retardation, difficulty following command requests, and expressive speech difficulty. As shown in Figure 1A, a prolonged EEG was performed, which showed intervals of generalized rhythmic 3-4 Hz activity of 11 minutes duration associated with absence seizure activity with prolonged duration and writing hesitancy. For Figures 1A-1B, we followed Hirsch et al. [6] on how this EEG was interpreted. In addition, generalized slowing and midline sharp waves forming 2-3 Hz periodic patterns were occasionally identified during wakefulness. Given findings on EEG suggestive of prolonged absence seizure duration and clinical symptoms of localized repetitive body movements of writing hesitancy, this pattern was most compatible with an atypical ASE. CT head and MRI brain were unremarkable, ruling out structural causes such as a tumor, abscess, or stroke. The patient had no signs of infection such as leukocytosis and a fever; therefore, infectious etiologies were also ruled out. The patient was diagnosed with ASE and received lorazepam 2 mg and levetiracetam 3,000 mg intravenously. The administration of antiepileptics showed EEG normalization and resolution of symptoms, as shown in Figure 1B. The patient received levetiracetam 1,500 mg twice daily and remained free of recurrent events for over 24 hours before discharge.

## Discussion

ASE is typically associated with a history of idiopathic generalized epilepsy with a younger age of onset and characteristic EEG findings [2]. Commonly, it presents as an abrupt halt in current behavior and is usually accompanied by a blank facial expression and impaired consciousness, referred to as "staring spells." These seizures are uninterruptible, and patients often have automatisms such as eye fluttering and lip-smacking [5]. In contrast to absence seizures, which last up to 10 seconds, ASE can last for hours to days [5]. Although an initial presentation with ASE is uncommon, there have been reports of it occurring in the pediatric population [7]. 

The literature surrounding absence seizures within the elderly population is sparse; risk factors or genetic conditions contributing to this are unknown. In our case, the patient did not have a history of dementia, head trauma, or brain tumors, which should all be ruled out as potential causes of ASE presentation. However, there was originally concern that our patient's presentation was psychogenic in nature. The presence of EEG abnormalities and repetitive focal movements effectively ruled this out and the diagnosis aligned with ASE.

It can be challenging to recognize ASE in older adults given that they are past the age of onset associated with idiopathic generalized epilepsy. Additionally, the predominantly negative psychomotor symptoms raise other differential possibilities that are more common in this demographic. Diagnostic clues typically include a prior history of generalized tonic-clonic convulsions [8] or cessation of benzodiazepines [9]. However, these were absent in the patient's case. The patient's onset occurred during the management of myelofibrosis, raising the possibility of late-onset de novo ASE as a complication of treatment. Seizures are a relatively uncommon but life-threatening complication after allogeneic stem cell transplantation, with incidence ranging from 1.6-15.4% [10]. Overall, patients with ASE have a poor prognosis and a lower quality of life; therefore, they warrant increased follow-up with physicians. Causes may include immune encephalitis caused by an unknown immune mechanism or reversible derangements such as electrolyte or drug toxicities [10].

Although the incidence of seizures after allogeneic stem cell transplantation is 1.6-15.4%, the prevalence is 7% with generalized seizures and focal seizures accounting for 46% of this [11]. ASE only accounts for 4.6% of the prevalence in both adults and children [11]. Additionally, most patients are seizure-free within the first year of treatment, as was shown in our patient. As ASE is difficult to diagnose in adults, it is important to highlight the fatality of such a complication in allogeneic stem cell transplantation patients. One study showed that the five-year survival rate was 71.4% in patients without seizures compared to 31.3% of patients with seizures post transplantation [11]. Although the potential reasons behind this phenomenon need more investigation, prompt diagnosis and treatment are vital and therefore require further recognition of ASE as a possible complication for adult patients with allogeneic stem cell transplantation.

## Conclusions

This case report adds to the rare body of literature discussing de novo ASE, whose clinical presentation can be indistinguishable from other behavioral conditions. We suggest prolonged EEG recordings in older patients with fluctuating mentation, as this is advocated for the diagnosis. For patients with EEG findings lying in the ictal-interictal continuum, additional evidence of clinical and electrographic improvement with antiseizure medications is required to establish the diagnosis. Moreover, its occurrence during oncologic treatment warrants clinicians to be on the lookout for similar presentations and encourage future reports of this condition in association with similar therapies. This case report provides value to providers treating patients with similar oncologic therapies and highlights the need for ASE to be further studied as it is a possible rare complication of allogeneic transplantation of stem cells.
